# Three-dimensional response of double anchored sheet pile walls subjected to excavation and construction sequence

**DOI:** 10.1016/j.heliyon.2019.e01348

**Published:** 2019-03-20

**Authors:** Massamba Fall, Zhengguo Gao, Becaye Cissokho Ndiaye

**Affiliations:** Department of Transportation Science and Engineering, Beihang University, Beijing, 100083, China

**Keywords:** Civil engineering, Structural engineering

## Abstract

In this study, a three-dimensional (3D) response of anchored sheet pile walls was investigated on double-anchored sheet pile system during soils excavation and tunnel construction sequence. This construction procedure is executed in areas front and adjacent the sheet pile walls. This paper focused on both areas of construction effects on the sheet piles. This numerical study aimed at the evaluation of the variation of bottom wall bending moment, top wall lateral and vertical displacements and anchor reactions forces exerted in the sheet piles. This paper also described the variation of the total anchor's reactions forces from the upper and lower anchors rows. A parametric effect such as upper and lower anchors rows distance was also performed to evaluate the variation of the wall bending moment, displacement and anchors total forces. The analysis results indicated that the reactions forces developed in the lower anchor rods are always higher than those developed in the upper anchor rods. The higher the distance between the upper and the bottom anchors the lower the displacement of the top wall in any stage of the construction. The minimum bottom walls bending moment is developed in the case where the distance between the anchor's rows divided by the wall height is 0.51. Positioning the upper anchors at 0.15 and the lower at 0.39 the wall height from the top wall will induce minimum top wall vertical displacement during soil excavation. This paper presents the results and findings of the parametric study performed.

## 1 Introduction

Retaining walls can be used to support back soil during excavation and construction sequence. The walls of sheet piles are widely used as a part of numerous structural designing activities, for example, earth retaining structures, braced cuts, cofferdams, and continuous walls of waterfront structures [1]. Retaining walls can be cantilever or Anchored sheet piles. In case of the wall, height is between 3 - 5 m cantilever sheet pile walls can be used [2, 3, 4, 5, 6, 7, 8]. In case of the wall, height exceeds 5 m or when the design consideration induces the limitation of the lateral wall deflection anchored sheet pile walls can be used [9]. Anchoring the sheet pile wall requires less penetration depth and less moment to the sheet pile because it will derive additional support by the passive pressure on the front of the wall and the anchor tie rod [10]. Seleem [11] showed the advantages of using a double-anchored sheet pile instead of a single anchored one using numerical simulation. It was found that a large reduction occurred in the values of maximum bending moments in the double-anchored system, in addition to a significant reduction in the values of anchor forces.

The tunnel construction sequence involves soils excavation to allow the tunnel boxes installation and connection follow by soils backfilling in the space between the boxes and the retaining walls [12]. This process will induce walls and surrounding soil displacement and stress variations [13]. Anchoring the walls will reduce the walls displacement and stress to achieve great serviceability. The prestressed anchor sheet pile wall is a kind of light retaining structure which has been widely used in the past twenty years. This structure has safety, reliability, less construction cost and, it has been successfully used in railway, highway and other engineering works. Also, the prestressed anchor sheet pile wall has proven to be an excellent aseismic structure from the investigation of damaged earth structures in the Wenchuan earthquake in China [14].

Numerical simulation can be a powerful tool to simulate the behavior of sheet pile during soil excavation and construction stage the monitoring values fit with the simulation values [15, 16, 17]. Bilgin [18] used 2D numerical simulation to study the behavior of sheet pile during the excavation of soils in front or backfilling of soils behind the wall. The study results indicate that walls constructed by the backfill method yield significantly higher bending moments and wall deformations. Xu [19] performed an analytical approach and numerical simulation to evaluate the stress distribution across a frictional-cohesive backfill behind a rigid retaining wall under static conditions. The two results were convergent. Seleem [20] performed a 2D numerical parametric study to evaluate the variation of maximum values of bending moments and anchor forces exerted in the sheet piles. The study results indicated that the forces developed in the lower anchor rods are always higher than those developed in the upper anchor rods. Gabar [21] deduced the higher value of maximum bending moment achieved at the stiffer sheet pile walls. Gabar [22] investigated the effect of soil and bedrock conditions below retaining walls on the wall behavior using 2D numerical simulation. The results show that the soil conditions below the wall, including the bedrock depth and the bedrock slope angle, may have a significant effect on the wall behavior and should be considered during the design of retaining walls. These researchers performed 2D numerical simulation usually not suitable for real behavior in a complex environment.

Luo [23] showed that the locations of excavation-induced maximum lateral wall deflection and maximum ground surface settlement using 2D numerical simulation. Three-dimensional (3D) analysis required much effort and time consuming to get results. In any numerical simulation analysis, we must evaluate the usefulness of 3D before beginning the calculation. Goh [24] studied the wall deflection in braced excavation using both two dimensional (2D) and three-dimensional analysis. The study shows that the 2D maximum wall deflections are much bigger than those for 3D. The results and the model approximation are more closed to those provide by the real engineering application. A validated 3D FE numerical model based on field measurements to investigate the effects of pile length, sheet's location, pile's stiffness (including pile cross-sectional dimensions and Young's modulus), and soil properties were performed by Tang [25]. The results showed that the sheet's location could affect the formation of soil arching and the mechanism of earth pressure distribution for sheet pile walls. Furthermore, the effect of the moment of inertia on the behaviors of sheet pile walls was significant, compared to Young's modulus of the pile. Also, the earth pressure behind and in front of a pile can hardly be influenced by the soil parameters. Bahrami [26] performed three dimensional (3D) modeling for excavation depths of 10–20 m to evaluate the effect of a penetration depth of a diaphragm wall on the behavior of excavations in sandy soils. The analysis revealed the safe values of penetration depth for designing a diaphragm wall. A wall penetration depth of 20% of the excavation depth in 10 m-deep excavation and a wall penetration depth of 40% of the excavation depth in 15 and 20 m-deep excavations were found as safe values for designing the diaphragm wall in sandy soil. However, these authors do not study the behavior of sheet pile walls in the construction sequence.

Sheet pile walls subjected to tunnel construction sequence can be regarded as a soil-structure interaction problem. Most of the studies focused on the interaction of the soil-pile under static condition. Many methods have been proposed to solve this problem. Conte [27, 28] imposed a shear-strength criterion of the Mohr-Coulomb type at the soil–pile interface. This criterion limited the shear strength at a value function of the normal force and the soil friction angle.

In this paper three-dimensional finite element analysis is used to simulate the tunnel construction process, sheet pile walls construction, prestressed anchor cables and excavation of tunnel foundation pit. Continuum model was used to simulate the soil-pile interaction during three-dimensional simulation. This study shows the development of displacements and internal forces on the double-anchored walls. It also describes the effect of adjacent soils excavation and tunnels part construction on the behavior of the sheet pile walls.

## 2 Background

### 2.1 Tunnel project

**Fig. 1:**
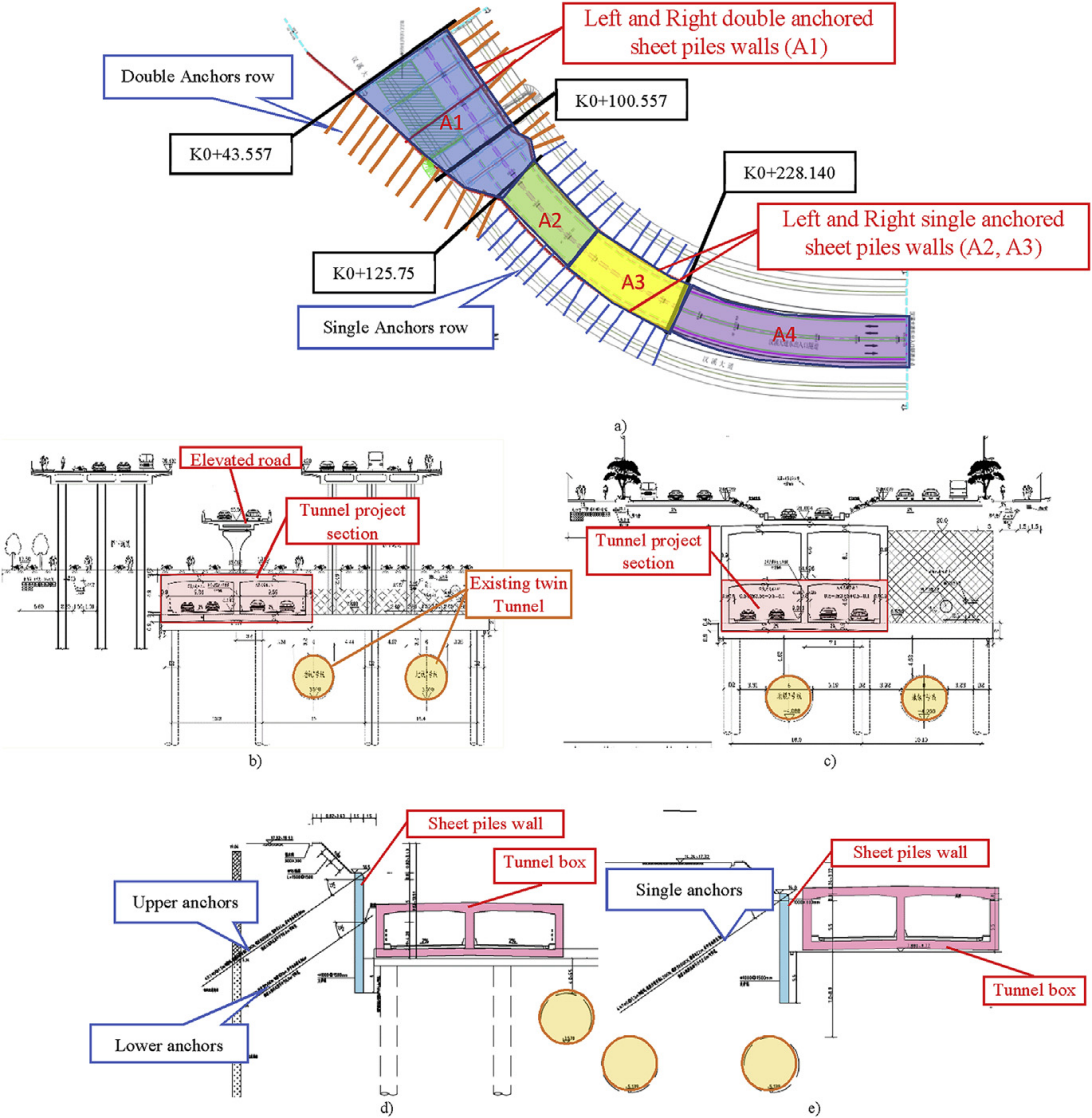
Section of the tunnel construction part and walls location, a) Relationship with tunnel project and existing structures, b) Project section at K0+43.557, c) Project section at K0+100.557, d) Sheet piles walls and steel anchors at K0+43.557–125.75, e). Sheet piles walls and steel anchors at K0+125.750∼ K0+228.140.

The tunnel construction processes involve excavation, which induces soils movement. Sheet pile wall can be used to support the soil from pouring after excavation (Fig. 1) shows the sheet pile walls position and the tunnel construction zone delimited in this project.

**Fig. 2:**
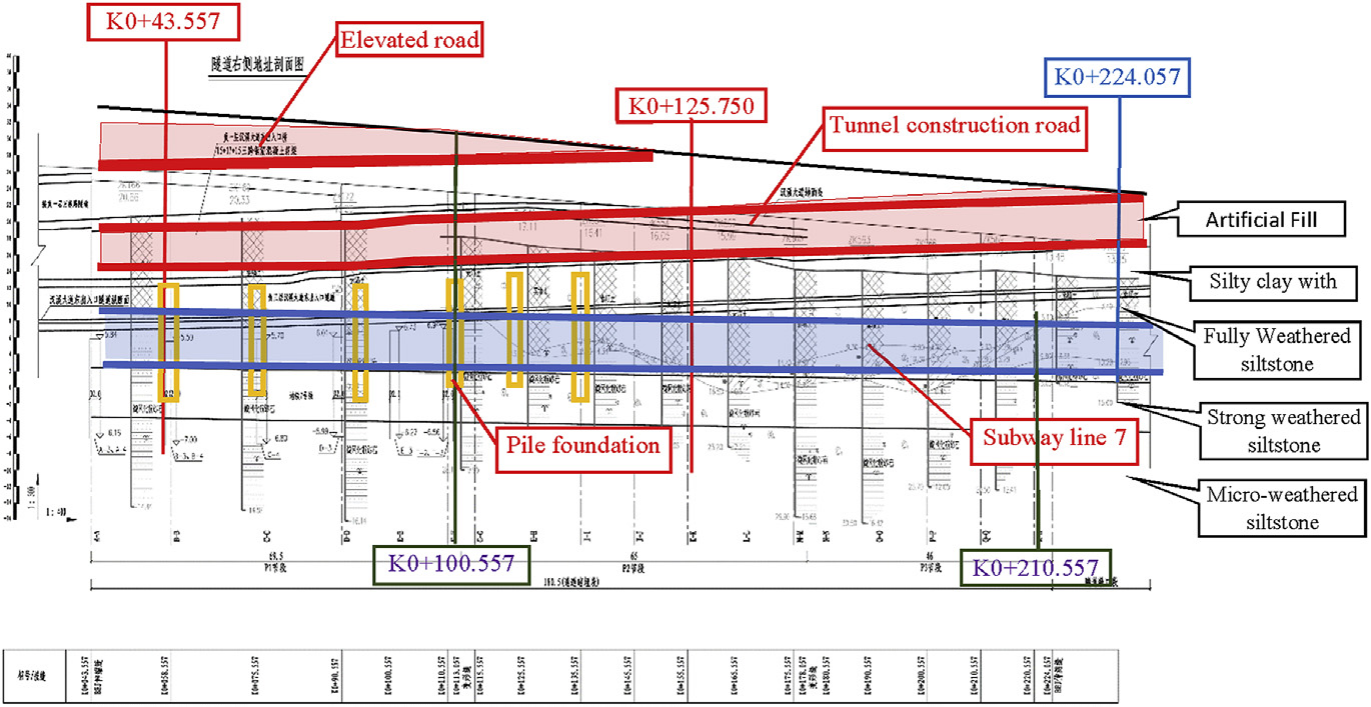
Tunnel Project Position and information's related to the project (soils layers, shape, etc).

The three-story tunnel runs from K0 + 043.557 ∼ K0 + 224 the closed section, 180.50 meters long (Fig. 2). The entire line set two-change slope point, the maximum longitudinal slope 2.296%. The three-story tunnel is a two-way circulation. Considering two-way four lanes, the single-hole standard section has a width of 9.35 meters and a partial increase to 9.62 meters with 0.8-meter wide inspection roads on both sides. The tunnel has a net height of 4.0–4.5 meters and a width of 3.5 meters. Tunnel entrance and exit position, the closed section entrance 0.3 × 0.55 meters interception ditches, along the longitudinal tunnel set 0.35 × 0.20 meters of drainage ditches. The tunnel water collecting wells are respectively arranged at the intersection of the closed section and the open section of each entrance.

The site is composed of five types of soil layers (Table 1). The soil parameters were obtained by carrying out laboratory tests. The 0 (zero) value is located at the top ground surface after the project backfilled. Many authors had studied the behavior of sheet pile walls in cohesion soil [29, 30]; several methods were proposed by the authors to solve the problem. In some cases, the method is compared with the equilibrium method commonly used in design to show the usefulness of the proposed method for practical purposes.

**Table 1:** Soils types and properties.

Soils Name	top layer (m)	Bottom layer (m)	Unit weight γ (kN/m3)	Young's Modulus (kPa)	Poisson's ratio	Friction angle φ (deg)	Dilation angle ψ (deg)	Cohesion yield stress (kPa)	Plastic Strain
Artificial Fill	0	-4.88	16.68	6.00E+04	0.3	20	15	5e5	0
Silty clay with	-4.88	-16.18	16.09	2.00E+05	0.3	23	19	5e5	0
Fully Weathered siltstone	-16.18	-25.21	16.19	1.20E+05	0.3	30	20	5e5	0
Strong weathered siltstone	-25.21	-62.82	17.66	6.00E+05	0.3	32	25	5e5	0
Micro-weathered siltstone	-62.82	-91.05	19.62	2.00E+07	0.22	35	30	5e5	0

### 2.2 Double anchored, wall and tunnel box properties

The left and right double anchored sheet pile walls considered in this study are 17.3m height and 0.3m thickness. The sheet pile is modeled a 4-node doubly curved thin or thick shell, reduced integration, hourglass control, finite membrane strains (S4R5). The material is concrete (Table 2). The sheet pile is assumed to be linear [31, 32] due to the complexity of the model. During the construction sequence, we remove and reactivate elements; this induces the use of a model change interaction between the sheet pile and the soil. Moreover, we do not use real contact to describe the interaction. The soil-pile interaction is described by the soil elastic plastic material model and constitutive law in contact zone (the pile is a concrete element more rigid than the soil; the interaction is described by the displacement inside the soil layers in contact zone). Young's modulus is decreased to 1% after the material yield in a tension state.

**Table 2:** Anchors and sheet pile materials properties.

	Sheet pile	Tunnel boxes	Anchor cable
Density (kN/m3)	24.525	24.525	68.67
Young's modulus (kPa)	3.5e7	3.5e7	2e8
Poisson's ratio	0.3	0.26	0.2

**Fig. 3:**
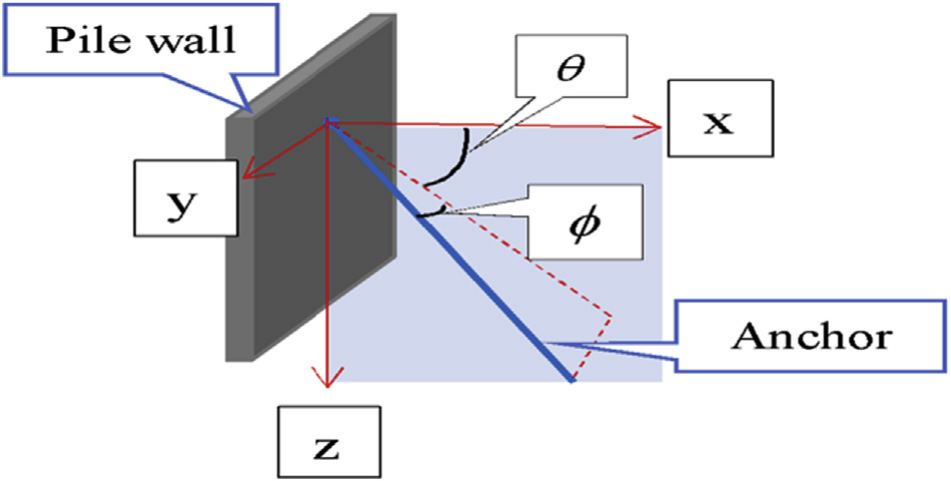
Three-dimensional angle used in this study.

Two layers of anchors with a vertical spacing varying during simulations were used for the support system. The length of the upper anchors ranged from 24 m to 30 m and for the lower anchors from 20 m to 27 m. The angle in three-dimensional space and the design parameters of the anchors used in this case are shown in Table 3 according to Fig. 3. The anchors are modeled with 2-node linear displacement (T2D2) to assume their behavior as truss element.

**Table 3:** Anchors spacing, length and slope.

Location		Anchor length (m)	Horizontal spacing (m)	θ(deg)	ϕ (deg)
Left Wall	Upper	24	2.84	35	0
	Lower	20	2.84	35	0
Right Wall	Upper	30	2.84	35	23
	Lower	27	2.84	35	23

**Fig. 4:**
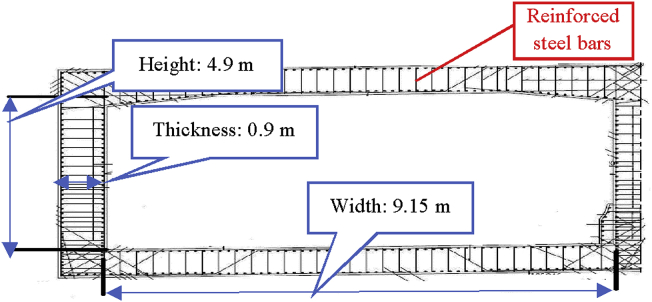
Typical tunnel box cross-section.

The tunnels boxes (Fig. 4) are modeled using linear elastic constitutive law, and the material is concrete.

### 2.3 Soil properties

All soils layers are modeled with 8-node linear brick elements (C3D8R) with reduced integration and hourglass control. The elasto-plastic behavior of the soil is simulated by using the elastic-perfectly plastic Mohr-Coulomb model available in ABAQUS/CAE. It is recommended to use this model for a first analysis of the problem considered. For each layer, one estimates a constant average stiffness. Due to this constant stiffness, computations tend to be relatively fast, and one obtains the first estimate of deformations. This Mohr-Coulomb model represents a 'first-order' approximation of soil or rock behavior. Besides the model parameters mentioned above, initial soil conditions, such as preconsolidation, play an essential role in most soil deformation problems [33].

## 3 Model

### 3.1 Numerical method

The effect of excavation, backfill, and tunnel construction procedure on the behavior of double-anchored sheet pile walls has been investigated through a parametric study: the distance between the upper and lower anchor rods.

**Fig. 5:**
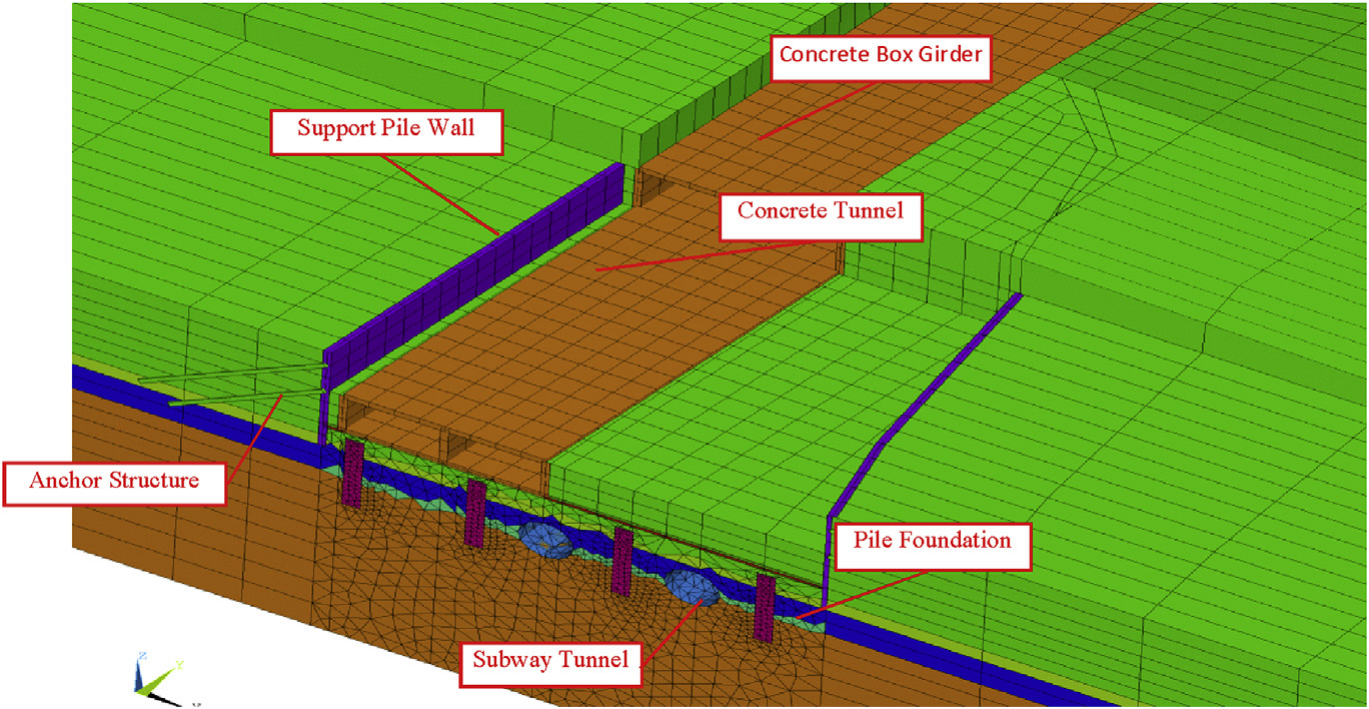
Three-dimensional finite element model of the structure.

The three-dimensional model calculated using the finite element method was carried out to evaluate the displacement, bending moment and reactions forces in any step of the construction sequence. Fig. 5 shows the three-dimensional model of the project. This calculation uses the large-scale general software ABAQUS/CAE. This software is widely regarded as the most powerful finite element software. It can analyze complex solid mechanics and structural mechanics, especially to control very large and complex problems and simulate highly nonlinear problems. It is widely used in the accurate modeling and analysis of various practical projects such as subways, tunnels, slopes, foundation pits, pile foundations, hydraulics, and mines, the field monitoring values fit well with the numerical results [34, 35, 36, 37, 38, 39].

The construction procedure is defined through the birth and death process because the material is added or removed from the system; certain elements in the model become come into existence or cease to exist. It's useful for analyzing excavation (as in mining and tunneling), staged construction (as in shored bridge erection), sequential assembly (as in the fabrication of layered computer chips), and many other applications in which you can easily identify activated or deactivated elements by their known locations.

Element loads associated with deactivated elements are zeroed out of the load vector. However, they still appear in element-load lists. Similarly, mass, damping, specific heat, and other such effects are set to zero for deactivated elements. The mass and energy of deactivated elements are not included in the summations over the model. An element's strain is also set to zero as soon as that element is killed. Several authors used successfully this method to define excavation and construction sequence.

The magnitude of the monitoring and analysis results can be generally considered similar [40, 41]. However, this method neglects the dynamic process of the construction plan. Including dynamic load induces by a driven process (piles, sheet pile) and by the excavation and construction processes. Some phenomena such as the dynamic stress redistribution, as well as the induced damage zone around the excavation under different lateral pressure coefficients could not be evaluated by this method. Zhu [42] revealed that the stress wave induced by the transient unloading would initially cause the damage only in the 1/3 radius vicinity of excavation perimeter. The damage zone may then develop further under the constant quasi-static far-field stress. The working load induces by the engines is also neglected.

**Fig. 6:**
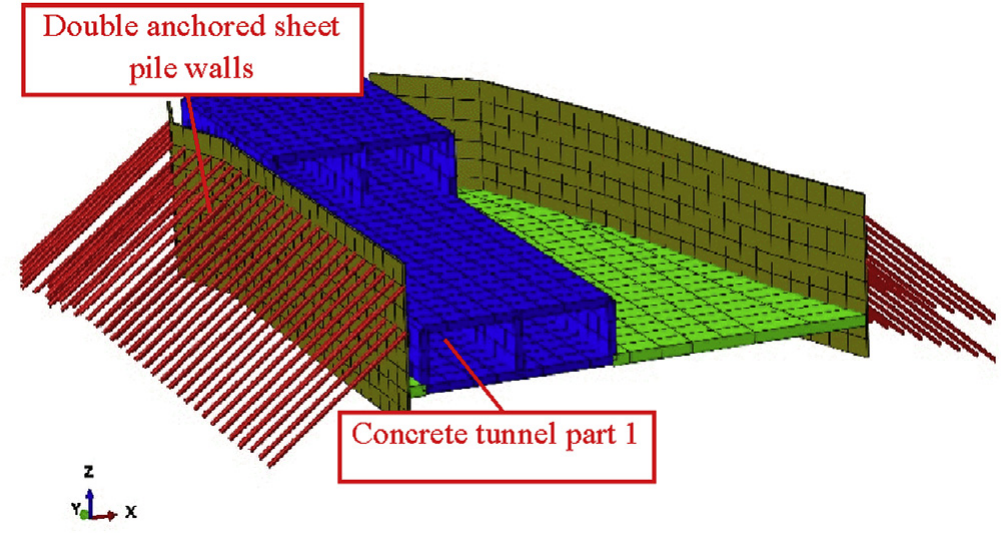
Double anchored and tunnel part in front of the wall.

**Fig. 7:**
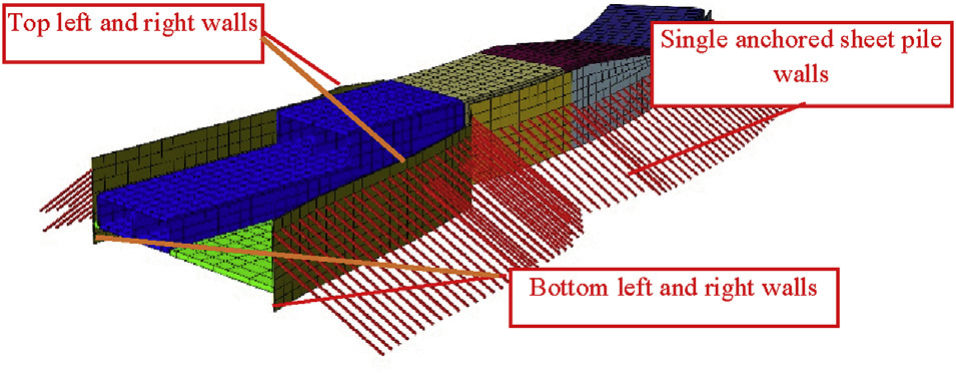
Anchors sheet pile wall and construction areas.

In this paper, we only focus on the effect on the double-anchored sheet pile walls due to the construction of the three-story tunnel. Fig. 6 shows the double-anchored sheet pile system with the tunnel part in front of. Fig. 7 shows de double-anchored sheet pile wall with the whole tunnel constructed, the adjacent single anchored sheet pile wall and the longitudinal top and bottom walls considered in this study.

### 3.2 Construction procedure

**Fig. 8:**
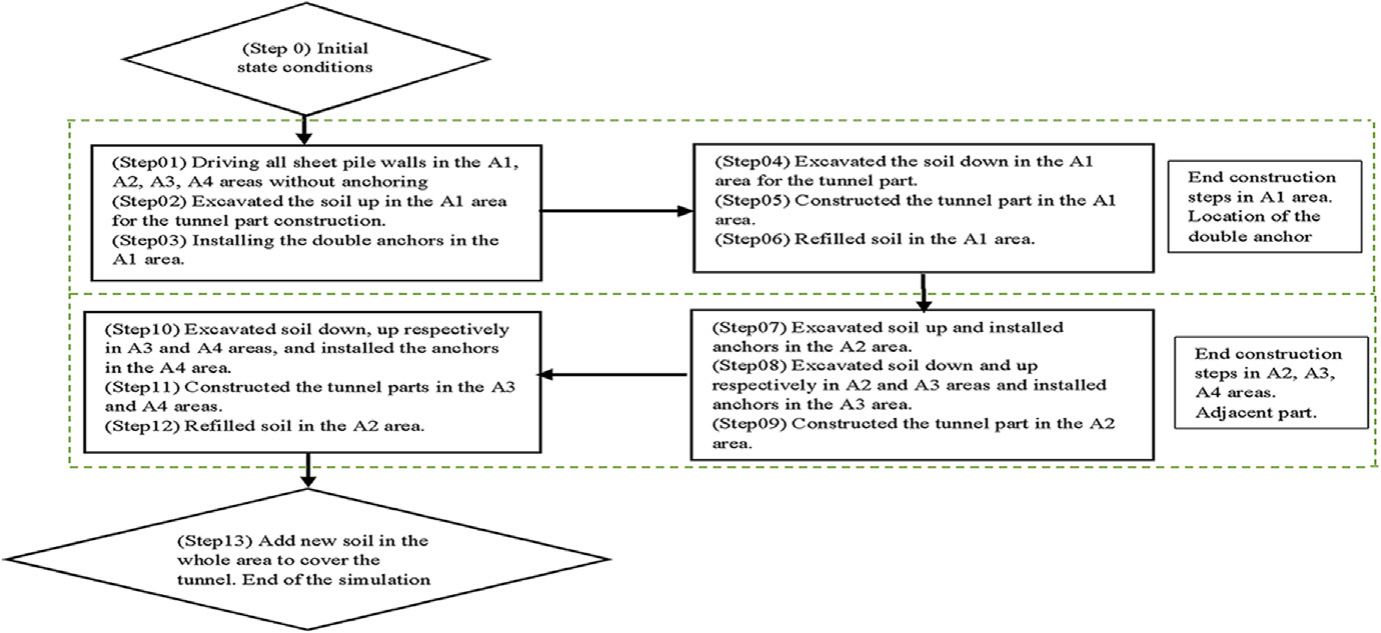
Tunnel excavation and construction sequence.

The construction procedure is separated into thirteen (13) steps preceded by the initial state calculations. Firstly, we evaluate the initial state conditions before beginning the construction procedures as defined in Fig. 8. Defining the tunnel construction plan involves optimization of the task during the construction to reduce the duration of the work and to minimize the displacement induce by each step calculations.

## 4 Results & discussion

### 4.1 Initial geo stress

While calculating the horizontal displacement, bending moment and anchors reactions forces of the sheet pile system. We need, Firstly to evaluate the site initial ground stress under the action of gravity. Secondly, remove the soil element during the simulation operation through the birth and death element, constructed the tunnel part and finally backfill in the working areas. The in-situ stress balance is performed according to the method of applying the initial stress and obtain the stress and displacement state of the model after the balance of the ground stress.

(The initial displacement rationality is zero. Due to the accuracy and complexity of the model, a small initial value should be calculated and the initial value used for calculation error).

In general, the initial conditions comprise the initial geometry configuration and the initial stress state. The initial water conditions for the soil layers also need to be defined. These conditions are also taken into account to calculate the initial effective stress state. Two methods are available to generate the initial stresses, gravity loading or the K_0_ procedure. The K_0_ procedure may only be used for horizontally layered geometries with a horizontal ground surface and, if applicable, a horizontal phreatic level. The value of K_0_ can be evaluated using Jaky's formula ${\text{K}}_{\text{0}}=1-\sin\;\varphi$ Where φ is the friction angle of the soil. In this paper, the complex shape of the soils layers will induce the use of gravity loading to generate the initial stress state.

This evaluation mainly uses numerical simulation to calculate and analyze stress and displacement predictions. This evaluation supposes that the soil and existing structures are already in a stable state. The results of this analysis are only the additional deformations caused by the implementation of the project on sheet pile walls.

The mechanical models used in the theoretical calculation of underground structures can be summarized into two types:

(1) Continuum model, i.e. soil-structure model; and
(2) action-reaction model, i.e. load-structure model.

These two mechanical models have their characteristics. The soil-structure model is mostly used for structural deformation analysis because of the interaction of stratum and structure. The load-structure model only uses the structure as the calculation object and is mostly used for structural internal force and deformation analysis. Specific to this project, considering the structural displacement of the sheet pile walls caused by the construction is closely related to the stratum, the soil-structure model is used for deformation analysis.

The dynamic load effect during construction processes on the sheet pile walls can be neglected according to the existing technical indicators for foundation pit excavation construction.

**Fig. 9:**
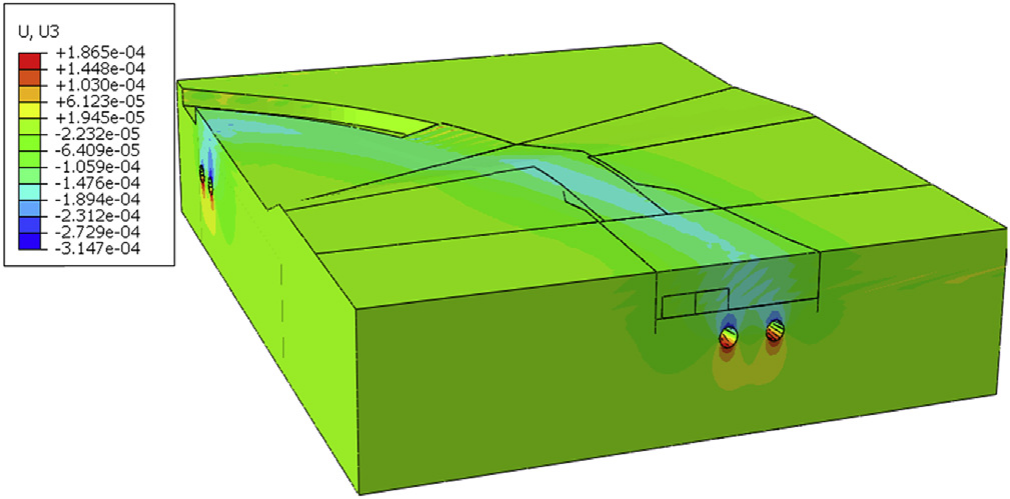
Initial vertical Displacement.

**Fig. 10:**
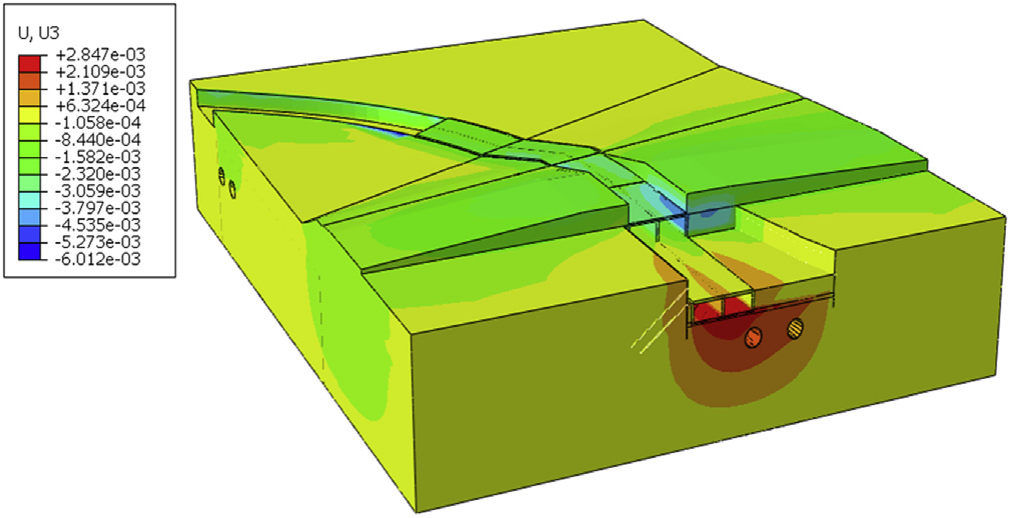
Vertical displacement after completion of the project.

After evaluation of the initial state results (stress and deformation), we begin the steps calculation all displacement of the model will be set to zero. Due to the accuracy and complexity of the model, a small initial value should be calculated and the initial value used for calculation error (Fig. 9). This will allow observing the additional displacement induces by each calculation phase overall model). After completion of the project, the maximum additional vertical displacement is 6.012 mm (Fig. 10).

### 4.2 Top wall lateral displacement

**Fig. 11:**
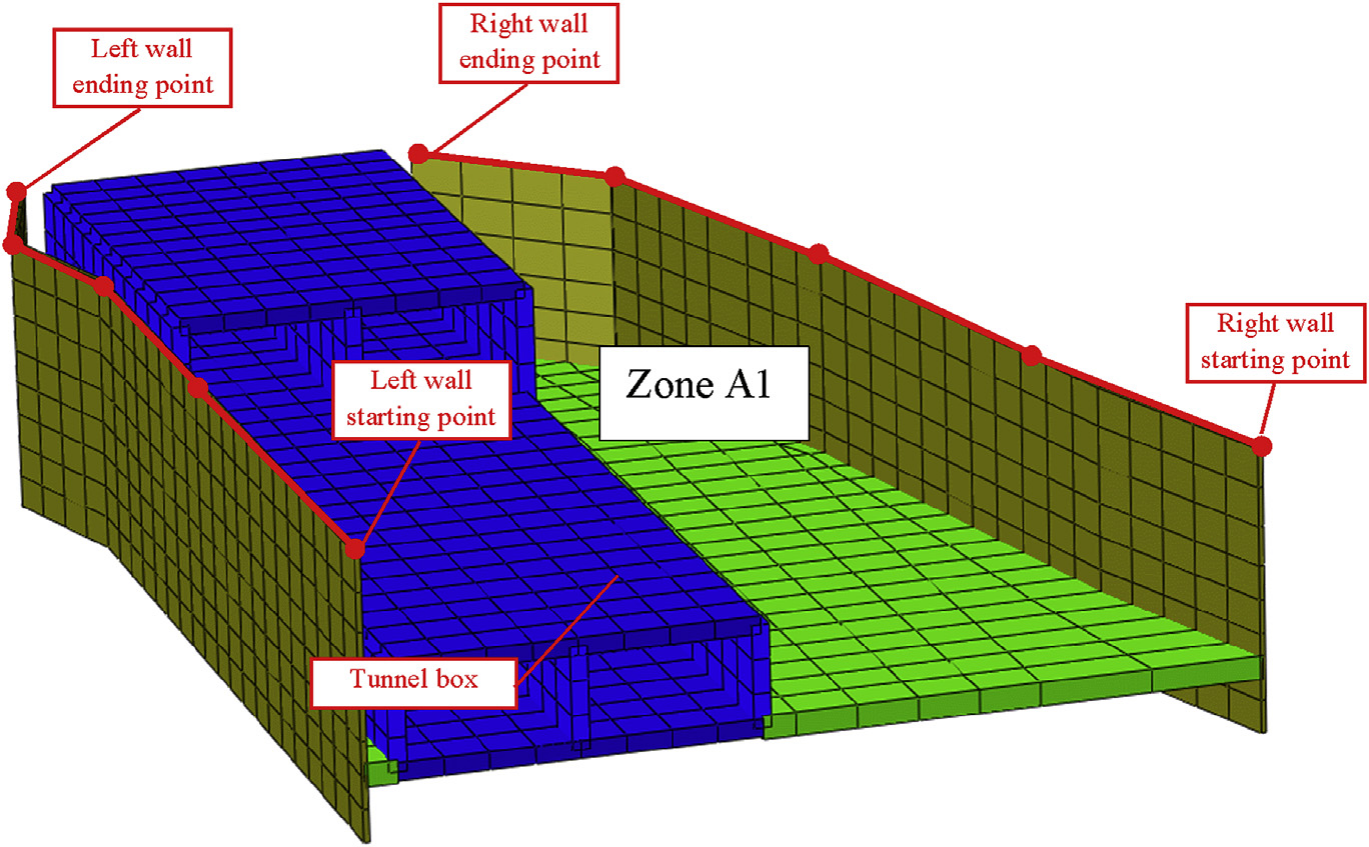
Top left and right walls path chosen.

**Fig. 12:**
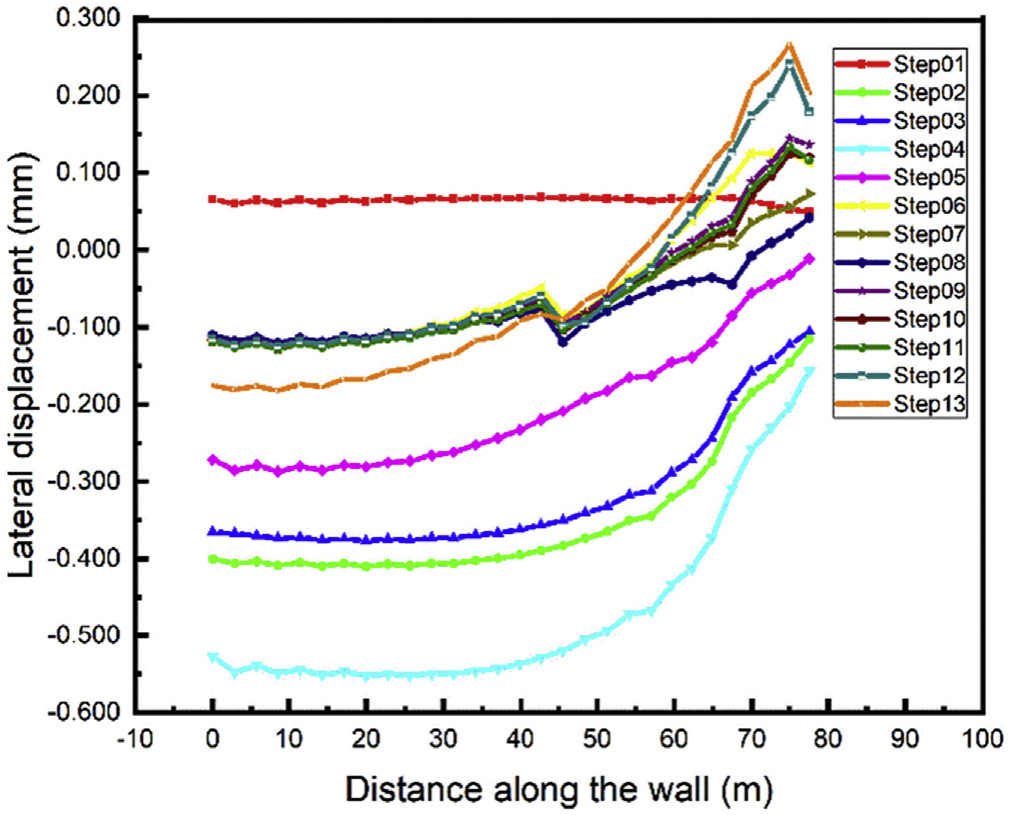
Left wall A1 lateral displacement.

**Fig. 13:**
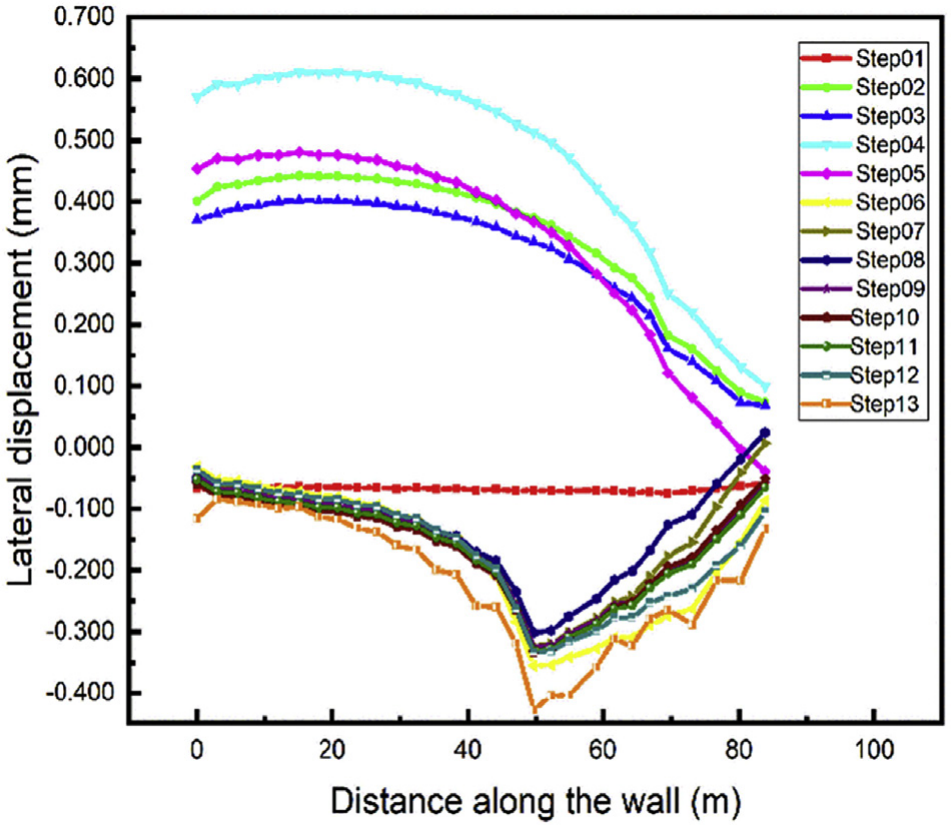
Right wall A1 lateral displacement.

Depending on the walls height, the tieback conditions, the soils properties the lateral wall displacement can be influenced. In this study, we focus on the double-anchored sheet pile walls to see its behavior during different load variations cases. The top left and right walls path chosen to evaluate the displacement is shown in Fig. 11. Figs. 12 and 13 show the displacement of the left and right walls after they had been driven in the soil is nearly zero (step01). During the excavation of the soil up in front of the wall large displacement occurs (step02) and tend to regain their initial position in step03 (installation of the anchors). The maximum horizontal walls displacement values appear during the excavation of the soil down (step04) and decrease during the construction of the tunnel part in front of the walls and the soil refilled in the A1 area (Steps 05 and 06).

The values almost stabilize in the construction steps adjacent the walls in the A1 area. The gap between the values in steps 07-12 decrease with the distance between the adjacent part and a point in the wall path increase. Backfilling the whole project area will have little impact on the top wall lateral displacement (step13).

We noted that the adjacent part construction has not many effects on the displacement of the anchored-sheet pile. In the following part of the work we will focus only on the area nearby the walls (area A1).

### 4.3 Top wall vertical displacement

**Fig. 14:**
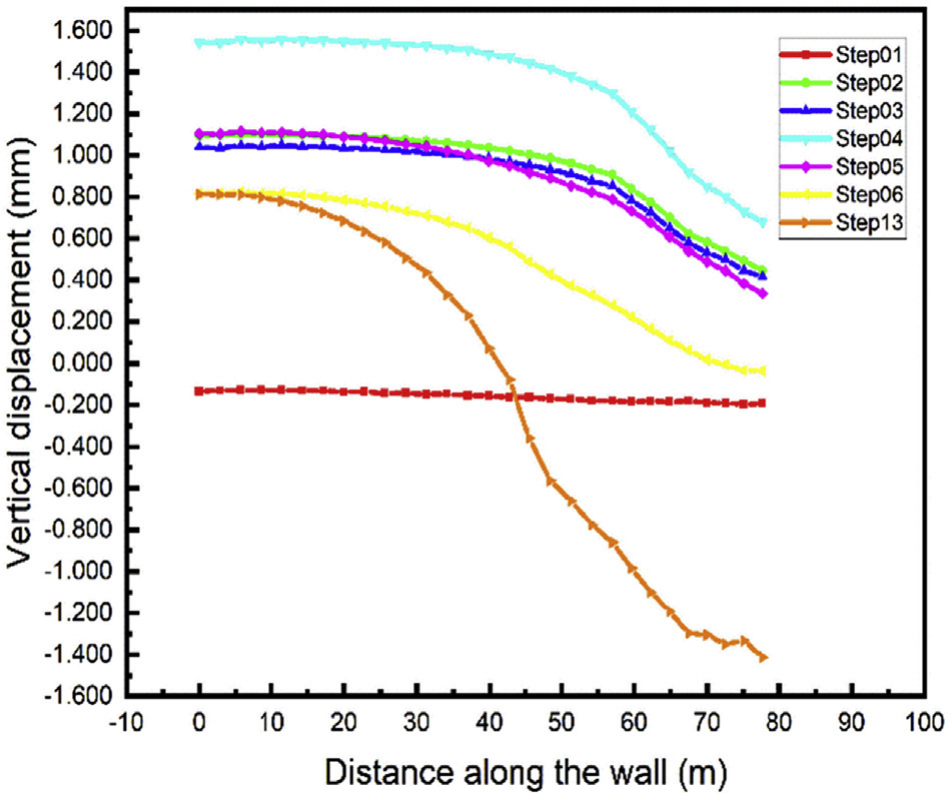
Left wall A1 vertical displacement.

**Fig. 15:**
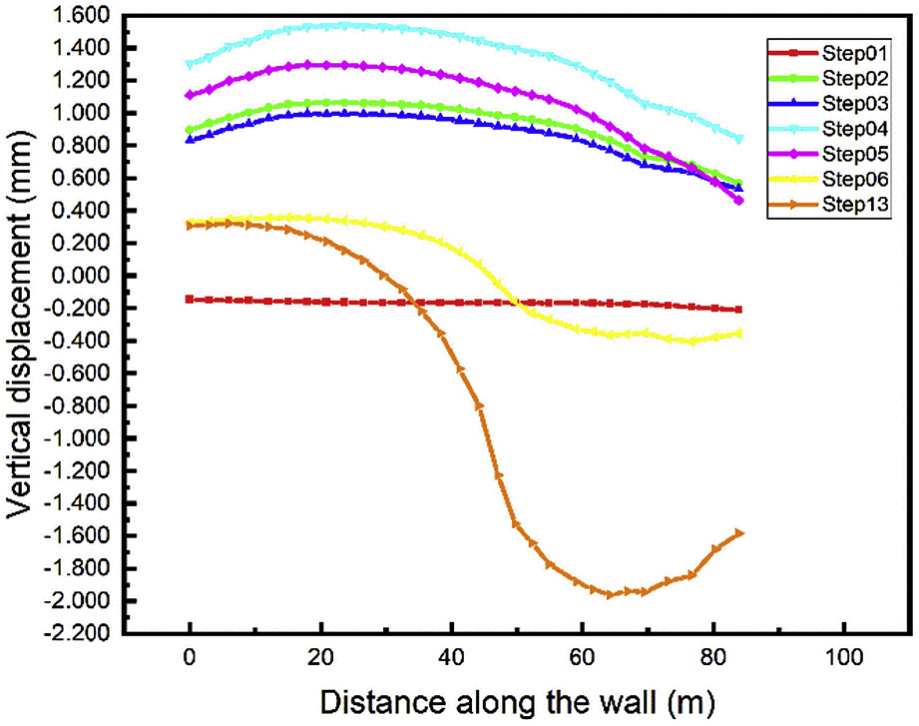
Right wall A1 vertical displacement.

Due to the maximum settlement values relatives to any structure, the vertical displacement of the top wall during all the stages of the construction can be observed. For both walls, the top initial vertical displacement is near to zero when the walls have been driven into the soil (Step01) Figs. 14 and 15. During the soil up excavation stage in step02 the top wall move above and the displacement value decrease during the installation of the anchor's cable (Step03). The maximum top wall vertical displacement in all steps is found in the excavation of the soil down in the A1 area (step04). The vertical displacement values decrease in the tunnel part construction and the tunnel part construction in front of the walls. Unpredictable top wall vertical displacement occurs during the whole project backfill stage (Step13).

### 4.4 Bottom wall bending moment

**Fig. 16:**
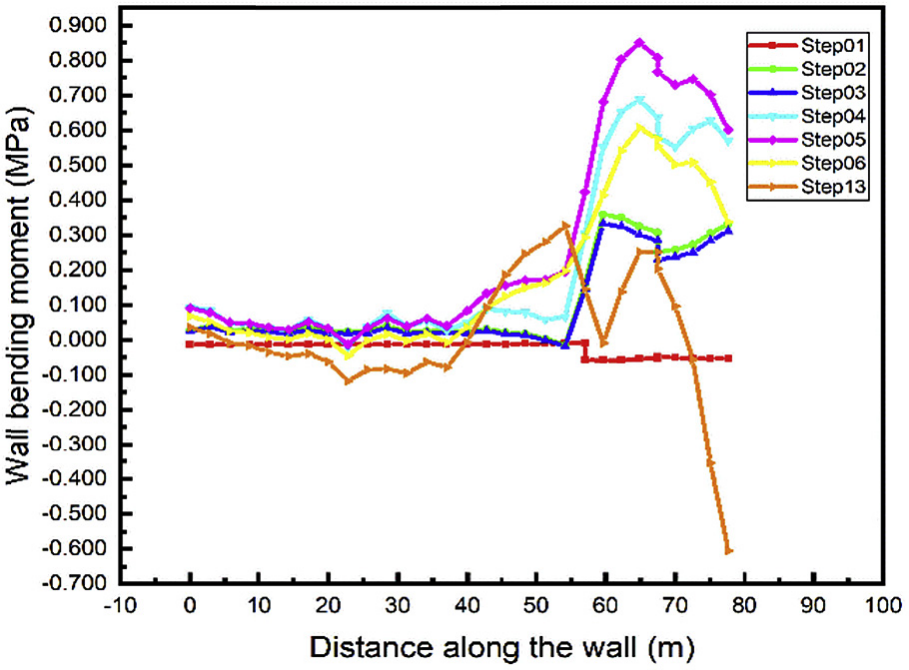
Left wall A1 bending moment.

**Fig. 17:**
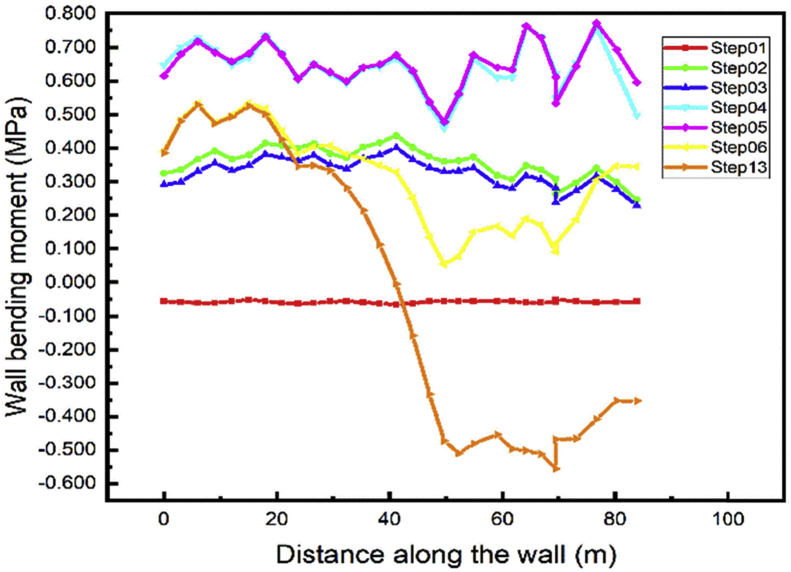
Right wall A1 bending moment.

Every material is subjected to maximum stress values that will induce rupture. Especially sheet pile walls bending moment is used for the design of this system of soil support. In this case, the bottom wall bending moment is investigated in different loads of cases induce by construction stages. Both Figs. 16 and 17 show the values of bending moment all along the bottom wall near to zero in the first step while the sheet pile is driven into the soil.

The bottom wall for both (left and right) is subjected to high bending moment during the step02 (excavation of soil up in A1 area), the values decrease a little during the installation of the anchored sheet pile in step03. Due to the excavation of the soil up in step04, the values increase again and reach their maximum in step05 (construction of the tunnel part in A1 area). The values decrease and almost stabilize in the construction steps adjacent the walls in the A1 area. The gap between the values in steps 07-12 decrease with the distance between the adjacent part and a point in the wall path increase. We notice unpredictable displacement in step13 due to the irregular shape of the backfilled soil.

### 4.5 Total anchors reactions forces

**Fig. 18:**
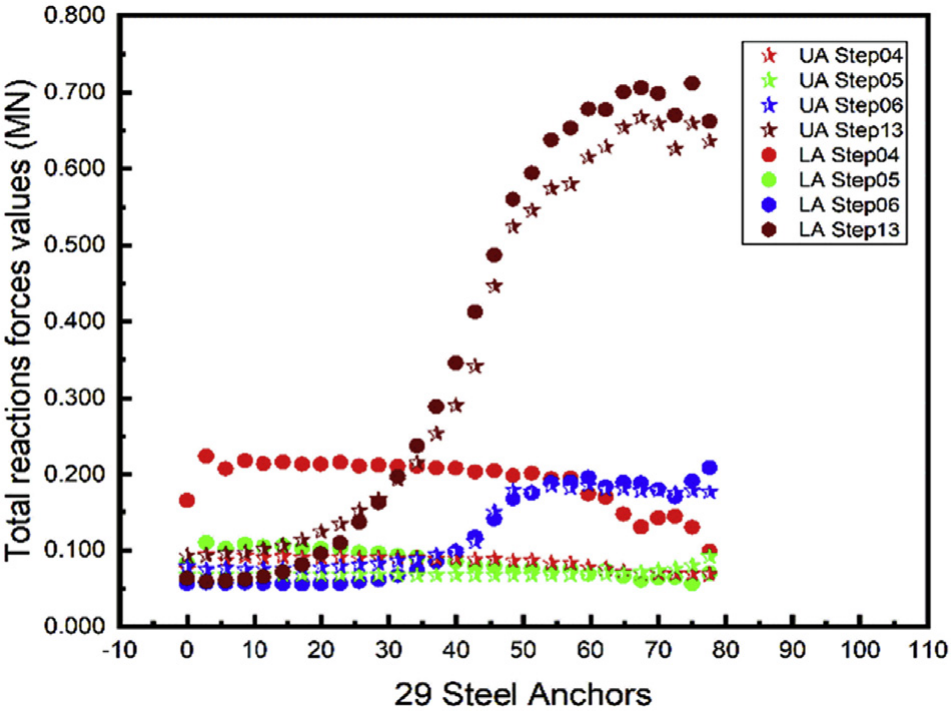
Left wall A1 reactions force.

**Fig. 19:**
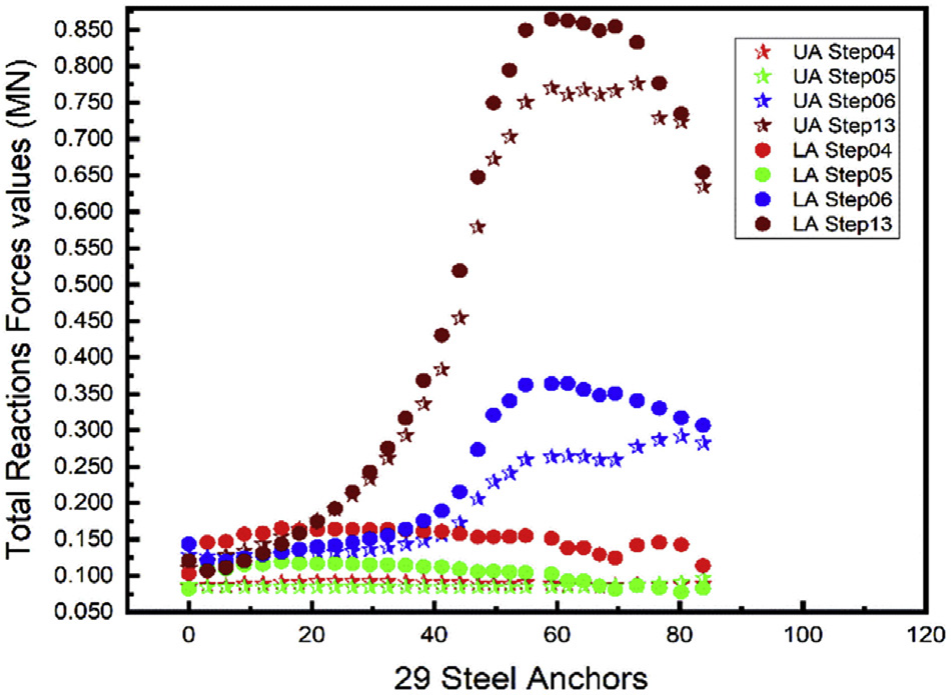
Right wall A1 reactions force.

Structural systems transfer their loading through a series of elements to the ground. The anchors are used to increase the stability of the sheet piles. The total reactions values are used for the design of the element that transfers the force to the ground at the fixed end of the anchors. Both Figs. 18 and 19 show the upper anchor's forces are always less than the lower one through all the construction stages. Similar results can be seen in [20]. The total anchor's reactions forces do not follow a predictable behavior during the construction steps. Higher reactions forces values occur during the whole project backfill (Step13). The design of the fixed end support in this project will consider the value obtain in the backfill stage.

## 5 Study area

The anchor's installation required the choose of the distance between the top wall and the upper anchor row, and the distance between the upper anchor row and the lower anchor row in case of a double-anchored system. Some authors used a numerical parametric study to see the effect of the distance between the top wall and the upper anchors in case of the single anchor system. This paper will focus only on the distance between rows anchors system in double anchored. A parametric value a evaluate as the distance between the anchor's row divided by the wall height will be chosen. The cases study is summarized in Table 4. The distance between the top wall and the upper anchor row is fixed through all the cases to 3.1m as a ratio of 0.15 the wall height.

**Table 4:** Parametric study values in all cases.

	a = Distance between anchor's rows/wall height
Case 1	0.27
Case 2	0.39
Case 3	0.51
Case 4	0.63

### 5.1 Effect on the top wall lateral displacement

**Fig. 20:**
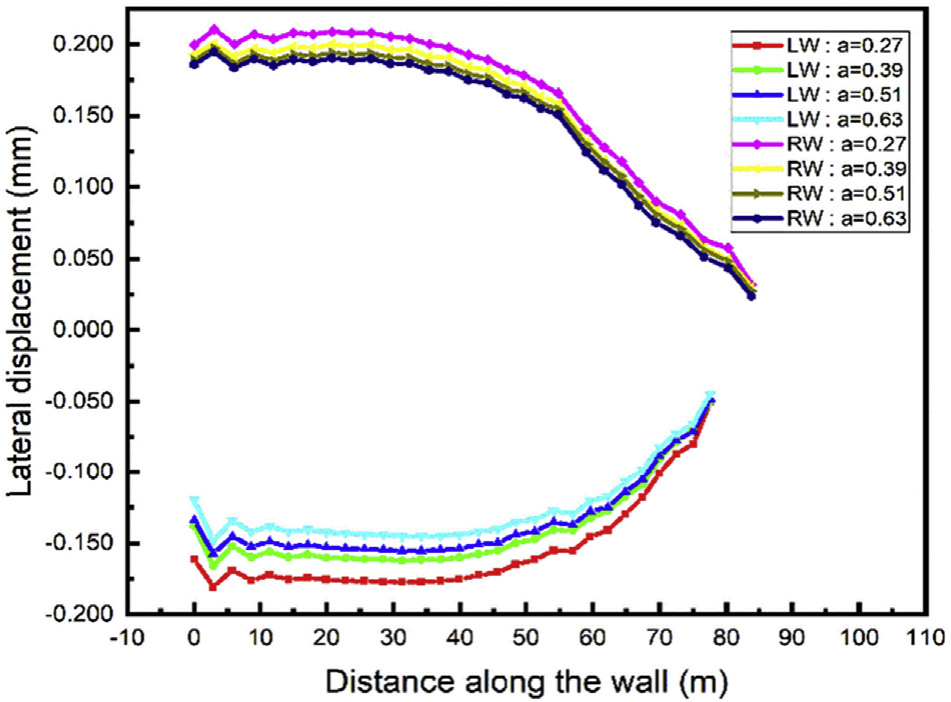
Lateral displacement of the left and right walls in step 4.

**Fig. 21:**
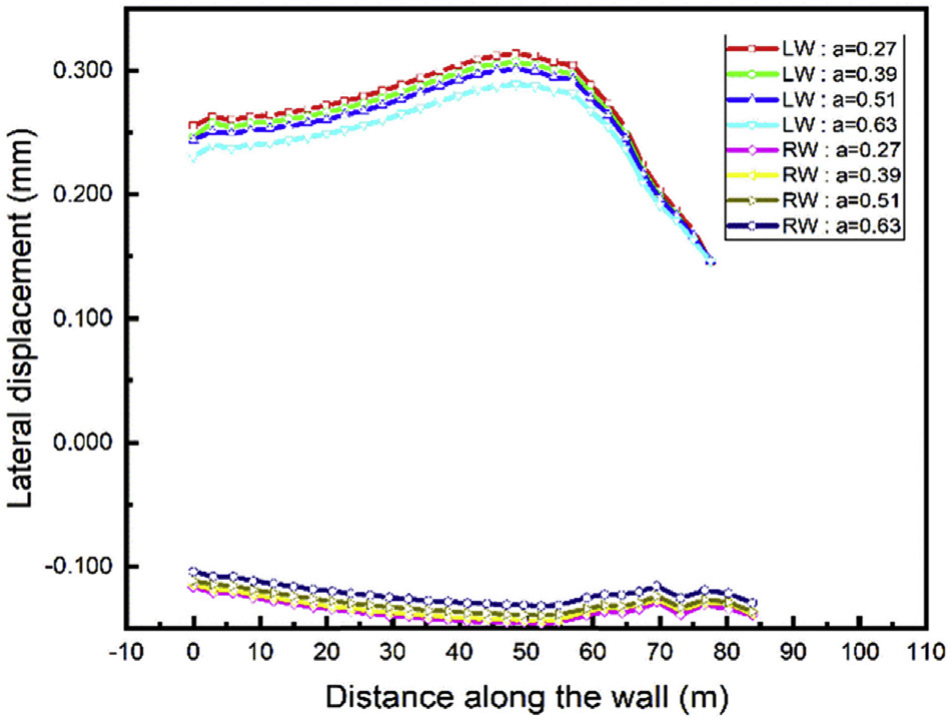
Lateral displacement of the left and right walls in step 5.

To study the effect of the anchor's row distance, the displacement value induces by each step calculation is more significant to draw some useful conclusions. In step03 begin the installation of the anchor cable, only step04 and 05 can be used to see the effects because in these steps large displacement occurs and they show the behavior during excavation (step04-soils removing) and tunnel construction part (step05-adding materials). The top wall lateral displacement has little variation during the adjacent tunnel sequence construction and the project backfill. Figs. 20 and 21 show that the top wall horizontal displacement value decrease with the distance between the anchor rows increase for both the left and right wall.

### 5.2 Effect on the top wall vertical displacement

**Fig. 22:**
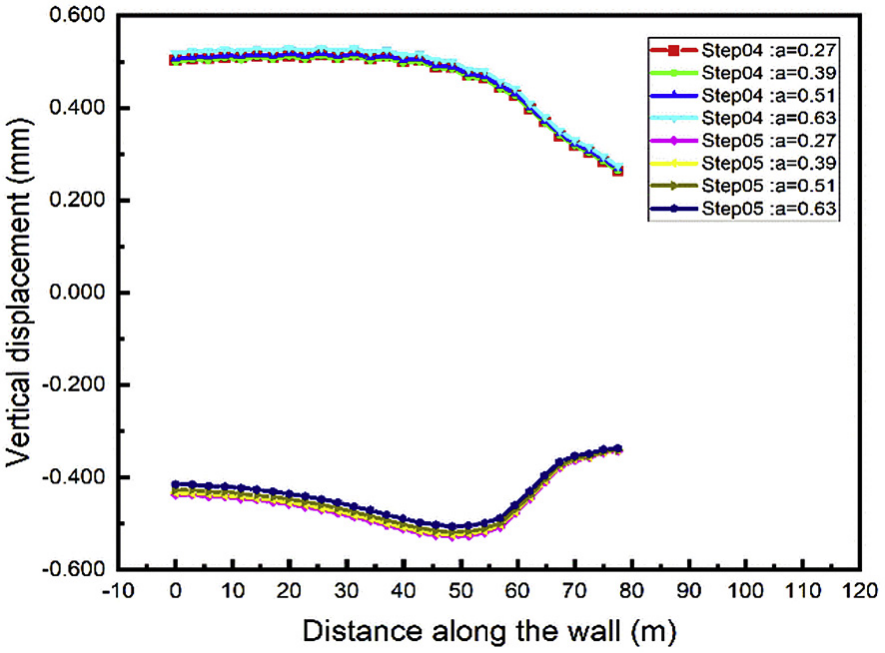
Left wall vertical displacement.

**Fig. 23:**
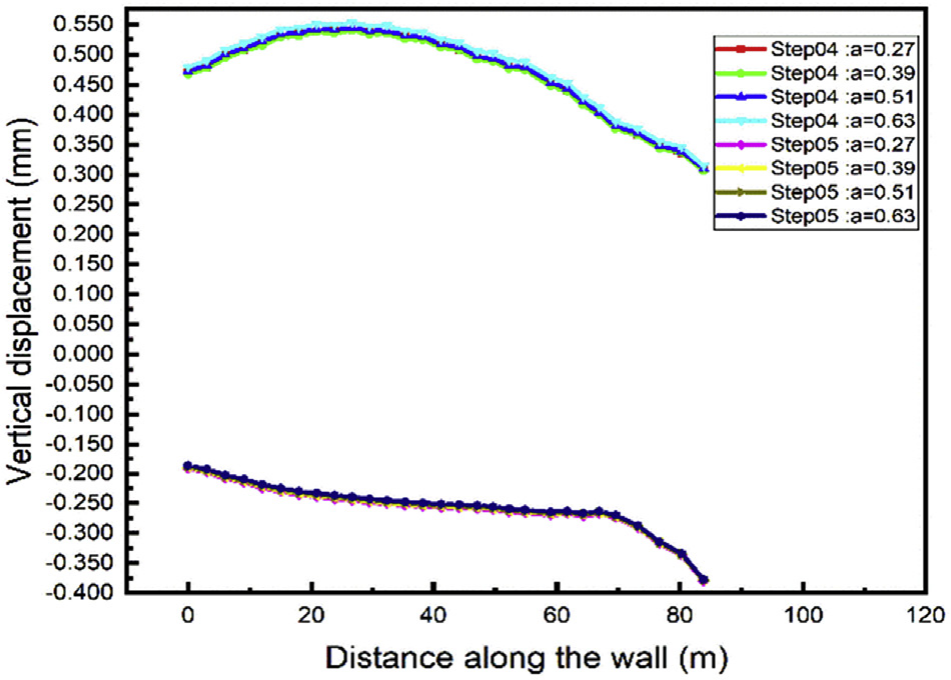
Right wall vertical displacement.

During the excavation process, the minimum top walls vertical displacement will occur during the case 2 while the distance between the two anchors rows is 0.39 the wall height Fig. 22. In the tunnel part construction, the top wall move down, and the value increase with the distance between the anchor's rows decrease Fig. 23.

### 5.3 Effect on the bottom wall bending moment

**Fig. 24:**
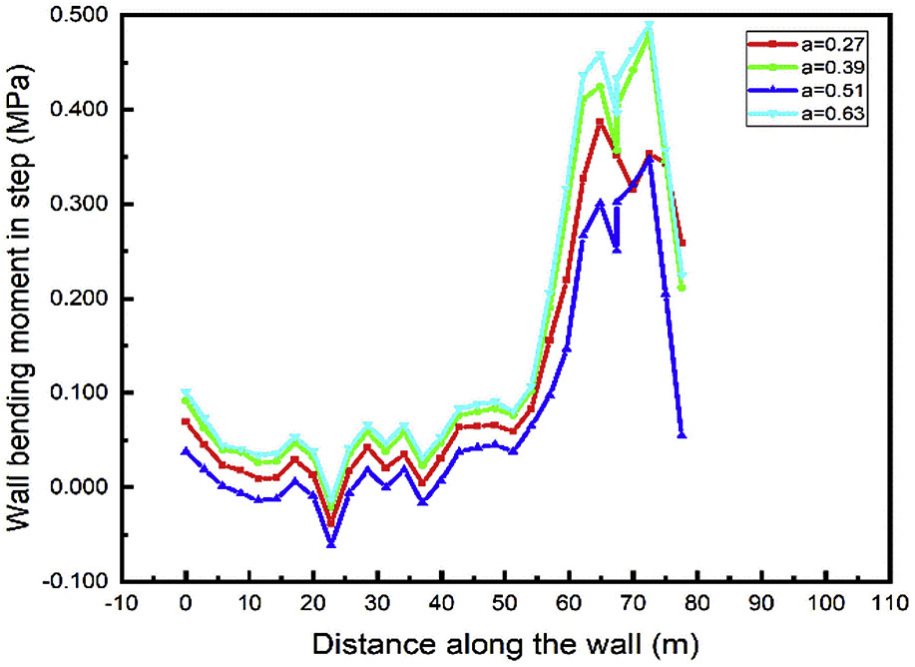
Left wall bending moment in step 4.

**Fig. 25:**
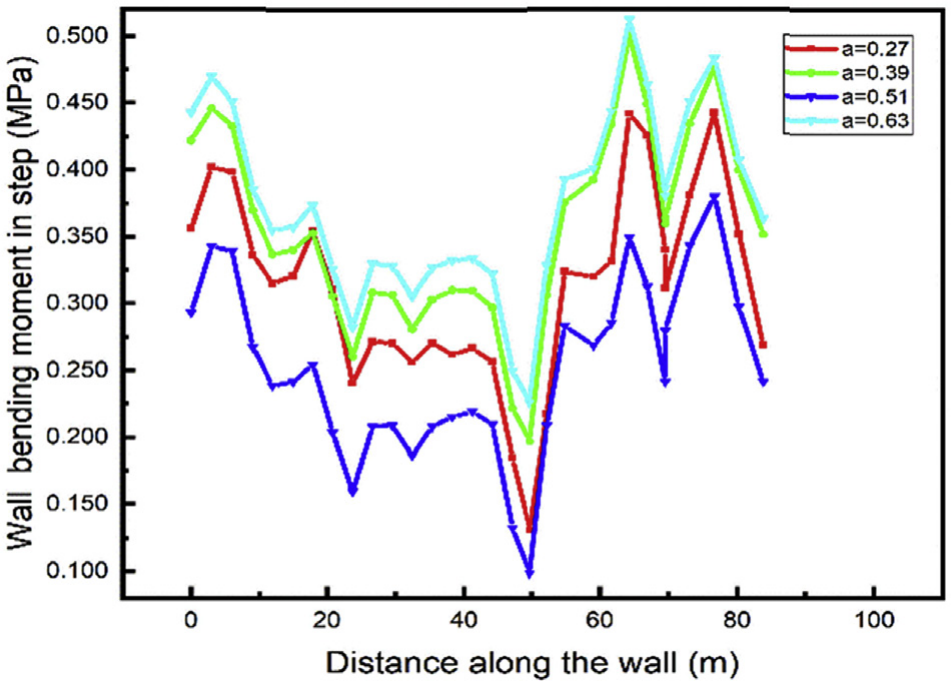
Right wall bending moment in step 4.

**Fig. 26:**
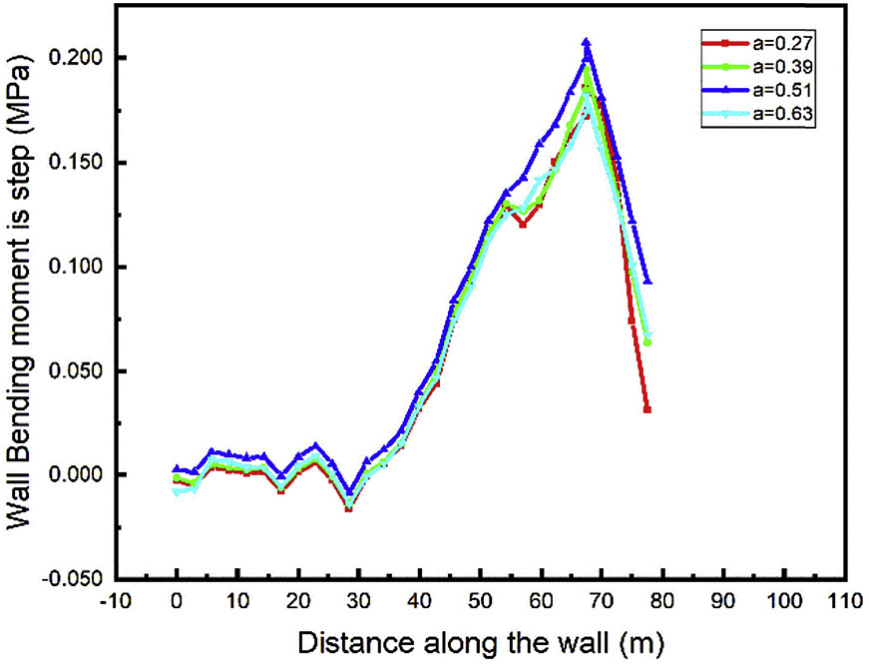
Left wall bending moment in step 5.

**Fig. 27:**
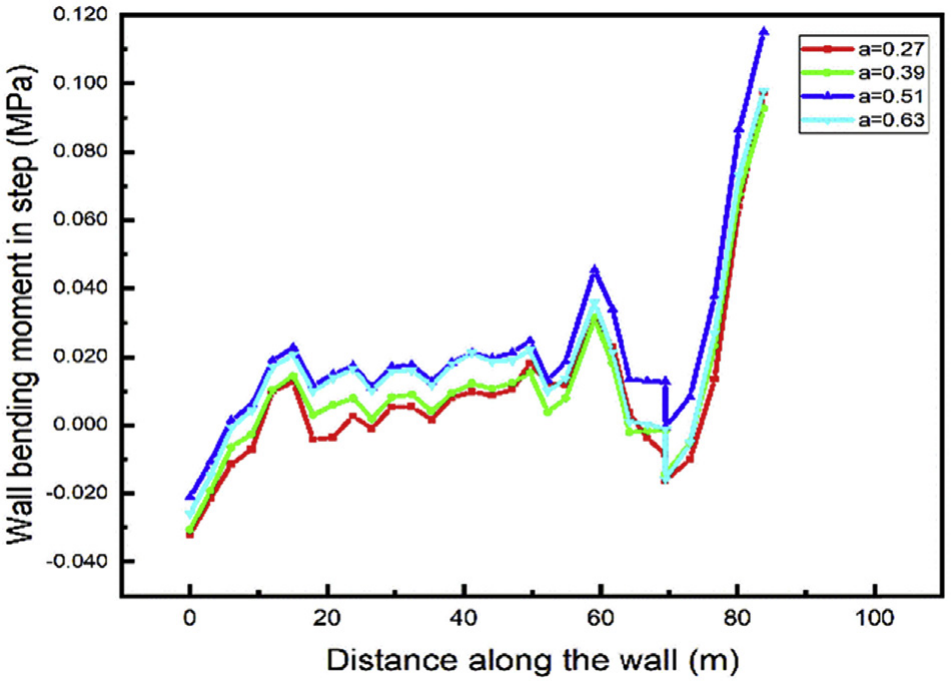
Right wall bending moment in step 5.

For both left and right walls, the lower wall bending moment during excavation (step04) occurs in case 3 and the maximum bending moment in case 4 (Figs. 24 and 25). In adding materials cases tunnel part construction (step05) the maximum value of the bending moment also occurs in case 3 (Figs. 26 and 27) and the lower value in case 1.

## 6 Conclusions

A numerical parametric study on both systems double-anchored sheet piles subjected to excavation, tunnel part construction, soil backfill was conducted to evaluate the variation of top wall horizontal displacement and bottom wall bending moment in the three-dimensional analysis. The conclusions below can be drawn:

❖ During the construction, sequence the top wall horizontal displacement decrease with the distance between the anchor's rows increase. Positioning the upper anchors at 0.15 and the lower at 0.51 the wall height from the top wall will induce minimum bottom wall bending moment during soil excavation and maximum value in soil adding (Tunnel part construction). Positioning the upper anchors at 0.15 and the lower at 0.39 the wall height from the top wall will induce minimum top wall vertical displacement during soil excavation. The values increase with the distance between the anchor's rows decrease during soil adding (Tunnel part construction).
❖ The adjacent part of the walls construction and excavation have little effect on the deformations of the top wall, the bottom wall bending moment and the anchors total reactions forces. The influence of the adjacent part construction decreases with its distance to the wall increase. The whole project backfill has little effect on the top walls lateral displacement in stage analysis calculation. Unpredictable top wall vertical displacement and bottom wall bending moment occur during the whole project backfill stage. It is recommended to evaluate the wall displacement and stress during project backfill to estimate its impact on the design of the sheet pile walls.
❖ The maximum bottom left and right wall bending moment occur during the tunnel box construction in the A1 area not during the excavation phase like the walls lateral and vertical displacement. While using double anchored sheet piles system, the reactions forces magnitude is higher in the lower anchors than the upper anchors in every step of the construction.

## Declarations

### Author contribution statement

Massamba Fall: Performed the experiments; Analyzed and interpreted the data; Wrote the paper.

Zhengguo Gao: Conceived and designed the experiments; Analyzed and interpreted the data.

Becaye C. Ndiaye: Performed the experiments.

### Funding statement

This work was supported by Beihang University.

### Competing interest statement

The authors declare no conflict of interest.

### Additional information

No additional information is available for this paper.
